# Modelling the Wind-Borne Spread of Highly Pathogenic Avian Influenza Virus between Farms

**DOI:** 10.1371/journal.pone.0031114

**Published:** 2012-02-14

**Authors:** Amos Ssematimba, Thomas J. Hagenaars, Mart C. M. de Jong

**Affiliations:** 1 Department of Epidemiology, Crisis Organization and Diagnostics, Central Veterinary Institute (CVI) part of Wageningen University and Research Centre, Lelystad, The Netherlands; 2 Quantitative Veterinary Epidemiology, Department of Animal Sciences, Wageningen University, Wageningen, The Netherlands; Northeastern University, United States of America

## Abstract

A quantitative understanding of the spread of contaminated farm dust between locations is a prerequisite for obtaining much-needed insight into one of the possible mechanisms of disease spread between farms. Here, we develop a model to calculate the quantity of contaminated farm-dust particles deposited at various locations downwind of a source farm and apply the model to assess the possible contribution of the wind-borne route to the transmission of Highly Pathogenic Avian Influenza virus (HPAI) during the 2003 epidemic in the Netherlands. The model is obtained from a Gaussian Plume Model by incorporating the dust deposition process, pathogen decay, and a model for the infection process on exposed farms. Using poultry- and avian influenza-specific parameter values we calculate the distance-dependent probability of between-farm transmission by this route. A comparison between the transmission risk pattern predicted by the model and the pattern observed during the 2003 epidemic reveals that the wind-borne route alone is insufficient to explain the observations although it could contribute substantially to the spread over short distance ranges, for example, explaining 24% of the transmission over distances up to 25 km.

## Introduction

Highly Pathogenic Avian Influenza virus (HPAI), Classical Swine Fever Virus (CSFV), and Foot-and-Mouth Disease Virus (FMDV) are highly contagious viruses affecting livestock and are among the World Organisation for Animal Health (OIE) listed diseases. The consequences of their recent epidemics in the Netherlands [1]–[3] have been enormous and include high mortality rates, economic losses incurred in implementing control strategies and reduced exports, and for HPAI, a risk of spread to humans [1], [4]. During the 2003 HPAI epidemic in the Netherlands, following detection of the first outbreaks in late February, movement bans were implemented followed by other control measures. Nevertheless, more farms became infected and therefore in the second week of March the measure of preventively culling contiguous flocks was adopted. In the end, 255 flocks were affected over the course of the epidemic and close to 30 million birds were culled; in addition, the virus was transmitted to 89 people causing one fatality [4]. Between 80% and 90% of the outbreaks occurred through untraced routes, with the farm infection hazard increasing in the vicinity of earlier infected (but as yet undetected) farms [5], [6]. The sustained between-farm transmission despite extensive control measures demonstrated the difficulty of controlling HPAI spread in poultry-dense areas.

The mechanisms underlying the between-farm spread of HPAI are not clearly understood, especially those of indirect transmission (involving vectors or fomites and possibly wind-borne transfer), as opposed to direct transmission (transportation of live animals between farms) [1], [5], [6]. Indirect transmission has played a major role in large epidemics involving viruses such as CSFV [7], [8] and FMDV [9]. In the analysis of the Dutch 2003 HPAI epidemic data, Boender et al. [5] used statistical spatial-temporal modelling techniques and identified high risk areas for epidemic spread. The same technique of using a spatial transmission kernel was used by [9], [10] in studies on the between-farm spread of FMDV in Great Britain. Although important insights, helpful for the development of control strategies laid out in contingency plans, were gained from these analyses, a lack of mechanistic (as opposed to statistical) understanding of the between-farm spread currently impedes the further improvement of these strategies. For example, the extent to which biosecurity measures on farms contribute to limiting indirect transmission is unclear, as is how these measures can be improved.

With stringent control measures put in place during epidemics including bans on the movement of animals, the direct spread of the virus is reduced. Therefore, indirect routes such as contamination of personnel and fomites do become the only pathway of virus spread. Indirect transmission could arise from human vectors transferring infective excreta such as manure from infected to recipient animals [11]–[13], mechanical transfer of excreta [6], [13], [14] or a possible combination of these mechanisms.

The need to determine whether wind-borne transportation of the virus is one of the untraced routes of HPAI spread between farms is apparent. The simplest way possible is that where the virus is transported by wind from an infected farm directly to an uninfected farm as has been considered in plume models for FMDV spread [15]–[19]. Otherwise, the dispersal may be through a multi-stage process. In such a process, the virus may be transported from infected animals to recipient animals by wind during certain parts of the route and by other means (for example humans and vehicles) on other parts. Both scenarios require quantitative insight into the deposition pattern of (contaminated) farm dust.

Davis et al. [20] conducted a study on the spread of Equine Influenza in Australia in 2007. They concluded that virus was spread over 1–2 km via wind-borne aerosols. However, the significance of wind-borne spread of HPAI is subject to divergent opinion. This lack of consensus was mentioned by Power [21], who also noted the absence of any testing to support or refute a wind-borne theory of HPAI spread during the epidemics in Italy and the Netherlands. This route is often considered insignificant, but with no serious underpinning based on quantitative evidence. For example, Swayne and Suarez [11] suggest that although aerosols and wind-borne contamination may have caused some secondary spread during the New South Wales HPAI H7N4 epidemic in 1997, they should not be regarded as important in the spread of infection. Yet in the analysis by Power [21] of the 2004 H7N3 AI epidemic in Abbotsford, BC Canada, air samples taken around the infected poultry houses confirmed the circulation of HPAI in the air outside the barns. This motivates our aim to quantitatively assess whether, and to what extent, this route may have played a role in the Dutch 2003 HPAI epidemic.

We do this by developing a model for wind-borne transmission of HPAI between farms, and comparing its predictions for the distance-dependent wind-borne transmission risk with the observed transmission risk in the Dutch 2003 H7N7 epidemic [5]. In our analysis, where possible, we use the Dutch 2003 H7N7 HPAI strain to quantify HPAI-specific parameters such as the within-flock basic reproduction ratio 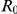. In our model, we consider the wind-borne dispersal and deposition of farm dust contaminated with HPAI. Our way of including deposition (that is, particle settling and accumulation on the ground) is in contrast to the existing plume models for wind-borne spread of FMDV and allows us to consider infection risks from inhalation by poultry of the originally deposited dust that becomes air-borne due to chicken activity instead of direct inhalation of air-borne dust arriving at ground level. We also include virus decay, as this influences the infection risks arising from deposited dust. Our model framework also allowed us to investigate dust deposition patterns between farms, which is relevant as a possible component of multi-stage indirect transmission mechanisms.

## Materials and Methods

In this section, we describe all the processes involved in the wind-borne spread of disease between two poultry farms. We start by modelling particle dispersion and deposition and proceed to determine the quantity of viable virus available in the deposited quantity. We then determine the distance-dependent risk of infection for farms downwind of an infected farm. Lastly, we compare our model estimates for distance-dependent probability of infection with a kernel derived from the Dutch 2003 HPAI epidemic data [5] that presents the averaged distance-dependent probability of infection.

### Dispersion model

Dust plume dispersion is assumed to originate from an elevated point source on a poultry house. A model of the motion and deposition of the (contaminated) dust plume is then used to calculate the quantity of viable virus in dust deposited at various locations as the plume moves. This model incorporates particle settling and pathogen decay and the principles of a 3D-Gaussian Plume Model (GPM) and assumes no barriers to the plume. This is a worst-case assumption for the Dutch situation since these barriers would reduce the distance covered by wind-dispersed particles.

The GPM used in this study was obtained by solving a simplified version of the general Advection-Diffusion (A–D) equation (Supporting Information S1). The classic GPM does not consider that during downwind motion the dust particles may settle down due to gravitational and other forces. However, we consider this process to be essential for two reasons: first, particle settling reduces the amount of dust moving further downwind, and second, we will be interested in the exposure of animals downwind to virus in settled dust. Hence, the first extension we make is incorporating particle settling, at a velocity *v*, into the classic GPM (Supporting Information S1). Particle settling leads to a shift, of magnitude 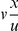, in the plume centre, where 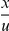 is the duration of plume flight. This gives the adjusted model as
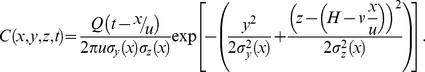
(1)Here *H* is the effective release height, *u* is the wind speed, 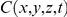 is the concentration of material at any location 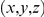 at time *t*, 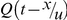 is the “mass flux” or strength of the emitting source, and 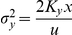 and 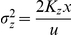, where 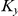 and 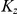 are respectively the lateral and vertical eddy diffusivities. The factor 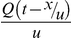 represents the total cross-sectional amount of dust per meter at a given location a distance *x* away from the source and 
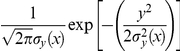
 and 
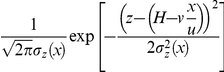
 are respectively the lateral and vertical dispersion components. Equation (1) was derived earlier (see Peterson and Lighthart [22] and Lighthart and Mohr [23]) and is used here as a starting point in the development of a calculation of the deposition pattern of the emitted particles.

### Deposition model

Particle deposition occurs as a consequence of the vertical plume expansion due to diffusion and particle settling due to gravitation. To model deposition, we first calculate the cumulative quantity deposited per square meter between the source and distance *x*, 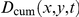 from the difference between the total quantity emitted and the part of the plume that is still air-borne at this point. Mathematically, this quantity is given by integrating the product of the total cross-sectional amount of dust and the vertical dispersion component in equation (1) with respect to *z* from negative infinity up to zero and multiplying it with the lateral dispersion component as
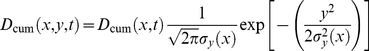
(2a)where
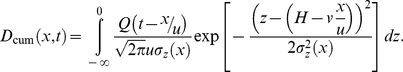
(2b)The quantity deposited per unit area per second at a specific point at a distance *x* from the source 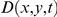 is now obtained from equation (2a) by taking the co-moving derivative 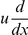 of the cumulative quantity 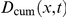 as
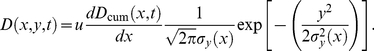
(3)An alternative way to calculate the total deposited quantity in a GPM, by integrating the vertical diffusion and settling rates of particles at ground level, is described in [24].

We then calculate the total quantity deposited per second on a rectangular area 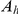 that is *2a* units wide (crosswind direction) and *2b* units long (downwind direction). If this area is always directly under the plume centre (that is, with no change in wind direction during the time of interest), we obtain this quantity by first integrating equation (3) with respect to *y* between the limits 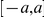 and integrate with respect to *x* between the limits 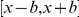. If we consider an off-plume-centre location 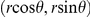 at distance *r* from the source farm and at an angle 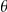 with the wind direction, the integration with respect to *x* is between the limits 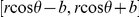 and the one with respect to *y* is between the limits 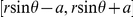 that is,
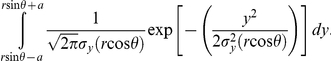
Carrying out the lateral integration explicitly yields the expression that estimates the total quantity deposited per second on an area 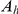 that is *4ab* square units, located at a distance *r* from the source as
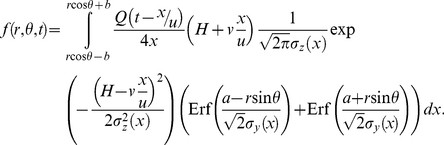
(4)


### Accumulation and pathogen decay

Consider virus particles emitted in a “puff” spanning a time interval 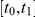 and decaying exponentially with rate constant 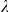. The accumulation and decay factor is obtained (see Supporting Information S1) as
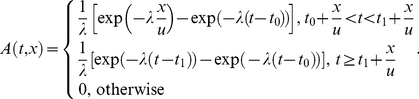
(5)It describes the accumulation of viable pathogen over time and gives the expected proportion of the particles that are still viable at time *t*. It takes into account virus decay during plume flight and while on the ground after deposition and its distance-independence is due to the fact that decay starts as soon as particles are released.

The total contaminated quantity 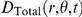 available at a given location 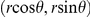 downwind after time *t* is obtained by taking the product of equations (4) and (5) as
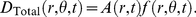
(6)Equation (6) defines the model for our study. In order to make a direct comparison of our predictions with the result of Boender et al. [5] which is a kernel describing the distance-dependence of transmission risk (averaged over all directions), we integrate the deposition function over all possible downwind directions and normalize the outcome. This gives the average contaminated quantity deposited as
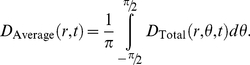
(7)This yields a fairly complex expression and thus the analytical insight obtained from it is limited. Therefore, most of the results discussed below are obtained by numerical analysis.

One question of interest is the distance from the source to the point of maximum deposition. This distance is calculated by solving the equation 
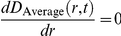
 for *r*, which again gives a complicated expression. Hence a numerical exploration of the effects of varying the model parameters is performed in the sensitivity analysis (Supporting Information S1).

### Estimating the distance-dependent infection risk for the receiving farms

#### Virus amount and infection probability models

To translate the predicted deposition of dust into virus amount we use results reported by Shortridge et al. [25] for the virus titer 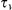 in originally wet faeces held at 25°C for 4 days. The log-transformed virus amount in 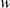 grams, 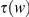 is given by
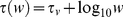
(8)in units of log_10_EID_50_. Subsequently, we determine the probability of infection of a chicken for a given virus amount inhaled based on a dose-response curve that we obtain by fitting to experimental data of Spekreijse et al. [26]. We use a dose-response function (probability of infection as a function of dose) as derived by Lange and Ferguson [27] based on assuming that there is a finite probability of infection for any virus amount even though the probability decays exponentially fast with reducing virus amount. For an inhalation involving 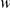 grams, the probability of infection 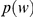 is given by
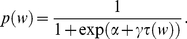
(9)where 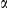 and 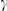 are the shape parameters for the fitted logistic curve. This dose-response function is consistent with the Independent Action Hypothesis [28].

#### The inhalation model

Chicken activities such as pecking, wing flapping, dust bathing and other movements suspend the already settled virus particles that they subsequently inhale. A study on determining the lung volume of chickens [29] reports a volume of 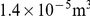
for a 24 days-old broiler chicken (which gives the limiting air sampling capacity 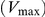 used in this study) and another study to determine the respiratory rate of chickens [30] reports a range of 27 to 31 min^−1^. Furthermore, since the components of farm dust which include faeces, skin and feathers, bedding material and feed-remains are not equally infectious, part of this material acts merely as a vector onto which the infectious part colloids during dispersal. The contaminated fraction 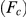 is taken to be 10% which is the relative amount of excreta in the litter attributable to chicken droppings [31]. More to that, the contaminated dust originating from infected premises is diluted on mixing with (initially uncontaminated) resident dust. The resulting composition of the dust to be inhaled is determined by scaling the quantity of the incoming contaminated dust by the amount of resident dust per unit area in a poultry house 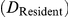 to obtain the fraction of contaminated dust in the total whirled-up dust. The concentration of inhaled dust is estimated by multiplying an estimate of the average dust concentration in a poultry house 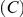 with a concentration ratio *c* describing how much the average concentration is exceeded closely above the ground. We use the average of the ratios of dust concentrations at 40 cm and 260 cm from [32]. Combining these model elements gives the weight of infectious material 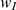 inhaled per inhalation as
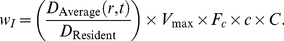
(10)


#### The within-flock epidemic model

Upon intake of the virus, infection may or may not occur depending on the virus amount inhaled. Given a successful first infection, subsequent infections at the farm level may occur, resulting into a major outbreak on the farm. In this study, infection risk is defined as the ability of the deposited virus to cause an infection of at least one susceptible bird in a flock and this bird being able to set off a major within-flock epidemic. For a flock with *N* birds, the hourly probability 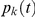
of infecting *k* birds is given by
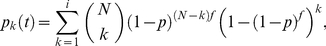
(11)where 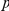 is the probability of infection per inhalation defined using equation (9) as 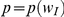.

For a disease which has a within-flock basic reproduction ratio 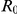 (defined as the number of secondary infections caused by a primary case in an entirely susceptible population), where for 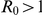, a major outbreak can occur, otherwise only a minor outbreak can occur [33], the probability of a major outbreak within the flock given *k* initial infections is 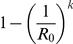. Therefore, the overall probability of infection of the flock 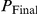 can be obtained from the product of the probability of having *k* initial infections and the probability that these infections cause a major within-flock epidemic as
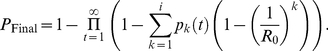
(12)We note that, unlike the deposition pattern, the probability of infection does not depend on available chicken space 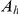. Rather, as described by equation (10), it is limited by the sampling capacity 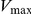 of the chicken.

#### Assessing the contribution of the wind-borne route

The contribution of the wind-borne route to the epidemic is here determined by the fraction of new cases that it can explain. We use the concept of the between-farm (basic) reproduction ratio (as defined in the Supporting Information S1) to compute this fraction.

#### Estimates for the parameters applicable to the HPAI situation

General and HPAI-specific parameter estimates are used in this study to quantitatively assess the possible role of the wind-borne route in the indirect transmission of the virus. They are categorised into dispersion, pathogen, host and farm related parameters.

Dispersion-related parameters include the emission quantity 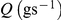 (which depends on the number birds on the farm, concentration of dust 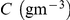 and ventilation rate 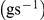), particle settling velocity 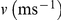, effective release height 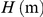, wind speed 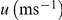, vertical and lateral eddy diffusivity 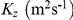 and 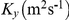 respectively. The total dust emission rate (both inhalable and respirable), taken from Takai et al. [34] is 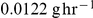
per bird and the total dust concentration is 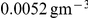. The average settling velocity for broiler house particles reported by Gustafsson and Mårtensson [35] is approximately 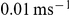. The effective release height was estimated as 6 m, based on the poultry house height of 5 m [32] and assuming an initial plume rise due to buoyancy of 1 m. According to Berge et al. [36], the vertical eddy diffusivity for outdoor plume modelling is 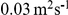.

The pathogen-specific parameters are the decay rate constant 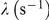 and dose-response parameters which depend on the combination of pathogen- and host-specific characteristics. For a virus that survives for 4 days [25], [37], the decay rate constant is calculated to be 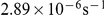. The host-specific parameters include the number of breathes per chicken per hour 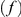 and the parameters that (through equation (9)) determine the probability of infection given an inhalation 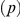. The farm-specific parameters include the flock size and the basic reproduction ratio 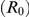. The estimate for the transmission rate parameter 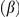 that Bos et al. [38] obtained using Dutch 2003 epidemic data is 4.5 per infectious chicken per day. To estimate the chicken infectious period 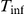, they used data from an experiment in which 7 out of 10 chickens died (resulting into 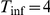
days), and the remaining 3 survived till the end of the experiment, here taken as 7.5 days that is, the average time between infection and depopulation during the outbreak [5]. In this study, these two pieces of information are combined to obtain a weighted average for the infectious period of 5.05 days and consequently, a within-flock 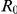 for the H7N7 HPAI strain of 22.7. For other strains of the virus, 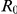 may be smaller, for example, it is estimated to be between 2.2 and 3.2 for the H5N1 HPAI strain [39]. Based on 7-days mortality data used in [38] and using a simple SIR model for within-flock transmission, we estimate that the reported mortality would correspond to an average number of infectious birds per day in a flock of roughly 100. To estimate the prevailing wind speed, we used data recorded at three weather stations ( two in the central and one in the southern part) in the Netherlands during the epidemic ( the period between February 28^th^ and May 31^st^ 2003) (available on the website: http://www.knmi.nl/klimatologie/daggegevens/selectie.cgi). From the downloaded data, we calculated the average (minimum-maximum) wind speed during the outbreak as 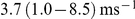. Here we use the average and perform a sensitivity analysis over the whole range (Supporting Information S1). A summary of all the parameter estimates is given in Table 1.

**Table 1:** Default parameter values used in the model calculations.

Parameter	Value	Source
Total dust emission rate, *Q*	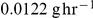 per bird	Takai et al. [34]
Total dust concentration, *C*	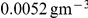	Takai et al. [34]
Concentration ratio, *c*	1.03*	Yushu and Baoming [32]
Log-transformed virus titer, 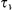	1.5log_10_EID_50_/gram	Shortridge et al. [25]
Particle settling velocity, *v*	0.01 ms^−1^	Gustafsson and Mårtensson [35]; Hinds [41]
Decay rate constant, 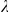	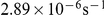	Webster et al. [37];
Shortridge et al. [25]		
Wind speed, *u*	3.7 ms^−1^	Meteorological data (KNMI)
Flock size, *N*	10,000*	Thomas et al. [6]
Effective release height, *H*	6 m*	Yushu and Baoming [32]
Eddy diffusivities, *K_z_* and *K_y_*	0.03 m^2^ s^−1^	Berge et al. [36]
Infection rate per day, 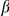	4.5 day^−1^	Bos et al. [38]
Weighted infectious period, *T*	5.05 days*	Bos et al. [38]
Basic reproduction ratio, 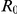	22.7*	Bos et al. [38]
Dose-response curve parameters 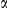 and 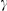	4.67 and −1.87*	Spekreijse et al. [26]
Area per hen (free range), 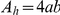	4 m^2^	EC [44]
Sampling capacity, 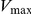	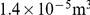	Julian [29]
Contaminated fraction, *F_c_*	10%	Koerkamp et al. [31]
Inhalations per hour, *f*	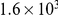 *	Pampori and Iqbal [30]
Resident dust amount per day, 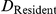	1.97 gm^−2^ *	Gustafsson and von Wachenfelt [45]

*parameter value estimated from the data in the indicated reference.

## Results

**Figure 1:**
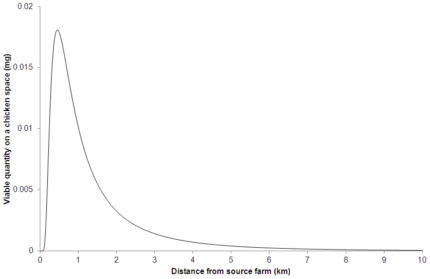
Contaminated dust quantity present on a 4 m square space at various distances from the source for the parameter values given in **Table 1** at the moment that the deposition arising from a 24 hour-long emission period ends.

**Figure 2:**
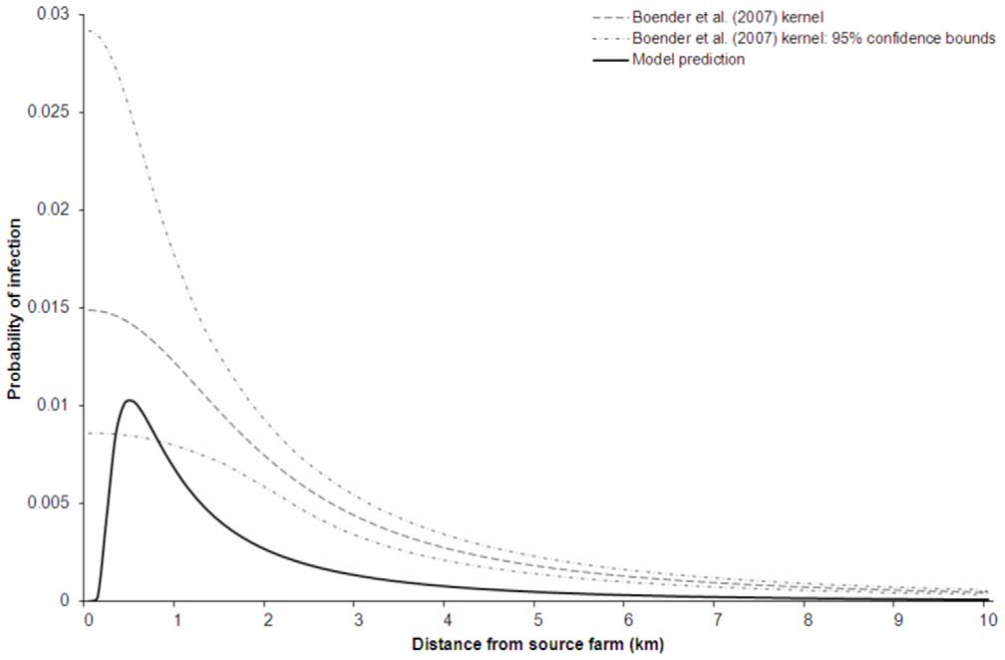
The distance-dependent probability of infection for the parameter values given in **Table 1** and the Boender et al. (2007) transmission kernel (and its 95% confidence bounds). The calculation caters for the prolonged infectiousness of the wind-dispersed material beyond the (direct-contact) infectious period of the source farm.

**Figure 3:**
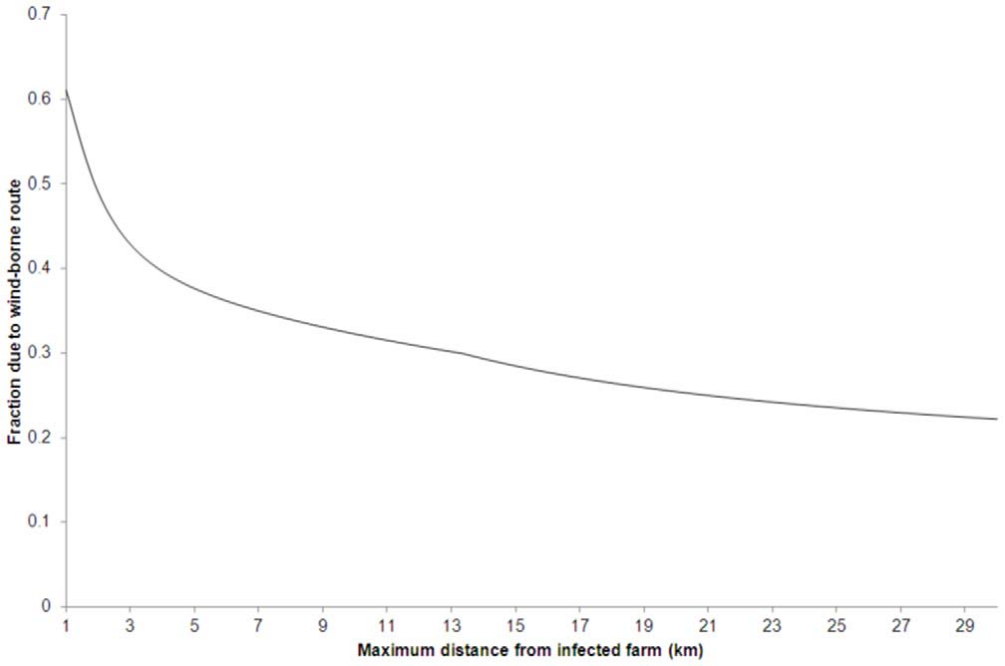
The fraction of the total number of new infections as estimated by Boender et al. (2007) from the 2003 epidemic data attributable to the wind-borne route for various choices of a cut-off distance up until which the new infections are occurring.

The model predictions presented here were obtained using the parameters given in Table 1 and the models given by equations (7–12). We present the model-predicted deposition pattern for contaminated dust in Figure 1, and in Figure 2 we show the comparison between the distance-dependent probability of infection as estimated by Boender et al. [5] from the 2003 epidemic data and our wind-borne spread model prediction. The fraction of new cases caused by the wind-borne route up until a given distance 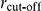 during the epidemic is presented in Figure 3. It is calculated for various choices of the cut-off distance 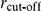. In the Supporting Information S1, we present detailed sensitivity analyses of the effect, on the deposition pattern, of varying; the wind speed (Figure S1), settling velocity (Figure S2), eddy diffusivity (Figure S3), effective release height (Figure S4), and decay rate (Figure S5). In Figure S6, we present the effect of varying the decay rate, the settling velocity and the within-flock basic reproduction ratio on the distance-dependent probability of infection.

### The predicted dispersal pattern of HPAI virus on dust

Following wind-borne dispersal of contaminated farm dust, we calculated the quantity of contaminated dust present on a given space (area per hen, 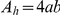) on an outdoor run of a farm. The predicted deposition pattern after a 24 hour-long emission is presented in Figure 1.

We observe (Figure 1) that for our choice of parameter values, there were no substantial quantities of contaminated dust present at distances less than 0.05 km from the source. This is because the model assumes that the particles are released through a raised vent (5 m above ground level). Beyond 0.05 km, the contaminated quantity present at a given location increased to its maximum at approximately 0.45 km from the source after which it starts to decrease. We use this result to estimate the distance-dependent risk of infection associated with the contaminated quantity present at a given location and compare the outcome with observed epidemic transmission pattern.

### Comparison with Dutch 2003 HPAI epidemic pattern

We calculate the distance-dependent probability of infection for farms downwind of an infected farm by combining our model predictions of the hourly depositions with the virus amount and infection probability models, the inhalation model and the within-flock epidemic model as described in the Materials and Methods section. We use the Dutch 2003 epidemic data to test whether wind-borne HPAI spread was possible and if so, determine its possible contribution during the epidemic by comparing our model predictions with the observed pattern in the epidemic. As can be seen from equations (7–12), for small infection probability per inhalation the model-predicted probabilities are to a very good approximation proportional to the deposition pattern (as given by equation (7)). As a result, in the parameter range of interest here, the distance-dependence of the model-predicted probabilities is practically indistinguishable from that of the deposition pattern.

The comparison in Figure 2 more importantly shows a qualitative difference in the tail. Compared to the observed pattern, there is a faster drop in the predicted infection probability beyond 0.45 km. At all distances from the source, the predicted probabilities are smaller than the observed risk. Also, beyond 1 km distance the predicted risk of solo wind-borne infection is decaying significantly faster with distance than the observed risk. The observed rapid decrease of the predicted risk with distance (Figure 2) is only very weakly sensitive to the precise value of pathogen decay rate, settling velocity and the within-flock basic reproduction ratio as shown in Figure S6. Based on these results, we conclude that the wind-borne route alone could not explain the pattern of the 2003 epidemic.

Figure 3 shows that the fraction of new cases that could be solely attributed to the wind-borne route decreases with increasing cut-off value 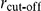. We consider the distance range of 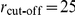 km to be most relevant as it corresponds to the width of the poultry-dense area in which the 2003 outbreak started [5]. Within this distance range, we estimate that the wind-borne route on its own could explain up to 24% of the new cases. Consequently, we conclude that the wind-borne route may have played a significant role in the spread of HPAI during the Dutch 2003 epidemic although it was not the only transmission route.

## Discussion

Quantification of the dispersal pattern of contaminated farm dust is of great importance in developing an understanding of the indirect transmission of livestock diseases between farms. In this paper, the quantity of viable virus deposited at locations downwind of a source farm is calculated using a GPM, and the significance of various model parameters to the deposition pattern is assessed. Based on our model predictions in the context of the spread of HPAI, the wind-borne route alone is insufficient to explain the observed pattern during the 2003 epidemic in the Netherlands. In particular, although it could have played a significant role in the shorter distance transmission events, it cannot explain the long-range transmission probabilities estimated in [5] from the observations in 2003. The calculation of the contaminated dust quantity deposited between farms could be a starting point for studies on multi-stage indirect transmission through a combination of different routes. This modelling framework can also be used to study the wind-borne spread of other pathogens.

In the sensitivity analysis (Supporting Information S1), we analysed the effects of varying wind speed (Figure S1), settling velocity (Figures S2 and S6), vertical eddy diffusivity (Figure S3), effective release height (Figure S4), the decay rate (Figures S5 and S6) and the within-flock basic reproduction ratio (Figure S6). The parameters to be explored were chosen based either on their importance to the dispersion process or on the uncertainty in estimating their values. A further reason for selecting the settling velocity and decay rate was to elucidate their importance in the study of wind-borne spread of livestock diseases, given that they are often neglected, for example in plume model studies of wind-borne spread of FMDV. The results of these analyses (Supporting Information S1) reveal the robustness of our main result. In other words, the discrepancy at farther away distances of the predicted risk and that observed during the epidemic as depicted in Figure 2 for the default parameter values of Table 1 holds for all ranges of parameter values explored. This is because, for all explorations, the resulting kernels have thinner tails compared to the pattern of the Dutch 2003 epidemic.

Since we were interested in assessing the role of wind-borne spread during the Dutch 2003 epidemic that involved an H7N7 HPAI subtype, we chose a within-flock basic reproduction ratio 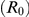 specific to this strain. However, for other strains such as the H5N1, the corresponding 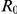 is smaller that is, in the range of 2.2 to 3.2 [39], and this consequently reduces the probability of a major within-flock outbreak although it is within the same order of magnitude as that predicted for the H7N7 HPAI virus strain considered in this study. Hence, we conclude that the predicted risk of infection by other virus strains at farther away locations will ultimately follow the same pattern as that of the H7N7 HPAI strain. Due to the lack of data on dose response and virus shedding for the H7N7 HPAI strain, we used data on H5N1 HPAI strain. However, the sensitivity analyses performed revealed that changes in these parameters do not alter the main conclusion of this study.

We conclude that the wind-borne route cannot fully explain observed patterns of between-farm spread of the virus especially for longer distances. This conclusion is robust to changes in uncertain model parameters. We also estimate that, up until 25 km distance, wind-borne transmission could explain up to 24% of the observed infections. This latter percentage is subject to some uncertainty. Nevertheless, this result supports the need to identify supplementary mechanisms that aid the transportation of the virus between locations. It also implies that: a) the experienced neighbourhood transmission was not entirely due to wind dispersal of the virus, b) virus transportation may either have entirely been by a different mechanism in a single-stage process, or c) virus transportation may have been by a multi-stage process that also involves the wind dispersal. Consequently, in-depth studies on the role of fomites in the transfer of infectious material between flocks are essential to develop alternative models for indirect transmission.

The deposition modelling approach developed here is likely to be relevant to modelling of wind-borne spread of other livestock diseases as well. Particles to which pathogens may be attached in wind-borne dispersal, have a size range of 1 to 100 µm and they sediment under gravity [40]–[42]. Therefore, it seems unrealistic to neglect the effect of deposition on the risk of wind-borne spread of livestock diseases. Also, it is important to incorporate pathogen decay when studying the wind-borne virus spread, especially for spread over more than just a few kilometres. For the case of FMDV this has previously been shown by Hess and others [43]. We have found, in the sensitivity analysis, that both deposition and pathogen decay have a significant effect on the ground level air-borne dust concentration at larger distances from the source (Supporting Information S1). These findings illustrate the general importance of considering the survival characteristics of the virus strain involved as well as the process of particle settling during plume motion if a reliable assessment of the risk of wind-borne spread of the livestock diseases is to be made.

## Supporting Information

Figure S1 **Effect of varying wind speed** ***u*** **on the contaminated dust quantity present on a 4 m square space at various distances from the source at the moment that the deposition arising from a 24 hour-long emission period ends.**
(TIF)

Figure S2 **Effect of varying the settling velocity** ***v*** **on the contaminated dust quantity present on a 4 m square space at various distances from the source at the moment that the deposition arising from a 24 hour-long emission period ends.**
(TIF)

Figure S3 **The effect of varying the vertical eddy diffusivity** ***K_z_*** **on the contaminated dust quantity present on a 4 m square space at various distances from the source at the moment that the deposition arising from a 24 hour-long emission period ends.**
(TIF)

Figure S4 **Effect of varying the effective release height** ***H*** **on the contaminated dust quantity present on a 4 m square space at various distances from the source at the moment that the deposition arising from a 24 hour-long emission period ends.**
(TIF)

Figure S5
**Effect of varying the decay rate** 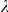 **on the contaminated dust quantity present on a 4 m square space at various distances from the source at the moment that the deposition arising from a 24 hour-long emission period ends.**
(TIF)

Figure S6 **Comparison of the distance-dependent probability of infection as estimated by Boender et al.** (2007) from the 2003 epidemic data and our wind-borne spread model prediction with default parameter values and: Panel A. The virus survival was increased from 4 to 7 days); Panel B. The particle settling velocity was reduced from 0.01 m/s to 0.005 m/s); Panel C. The within-flock basic reproduction ratio was increased from 22.7 to 100.
(TIF)
