# Supplementary figures and images for: Modelling the Wind-Borne Spread of Highly Pathogenic Avian Influenza Virus between Farms
Source: PLoS One. 2012 Feb 14;7(2):e31114. doi: 10.1371/journal.pone.0031114 (PMC3279517; doi:10.1371/journal.pone.0031114)

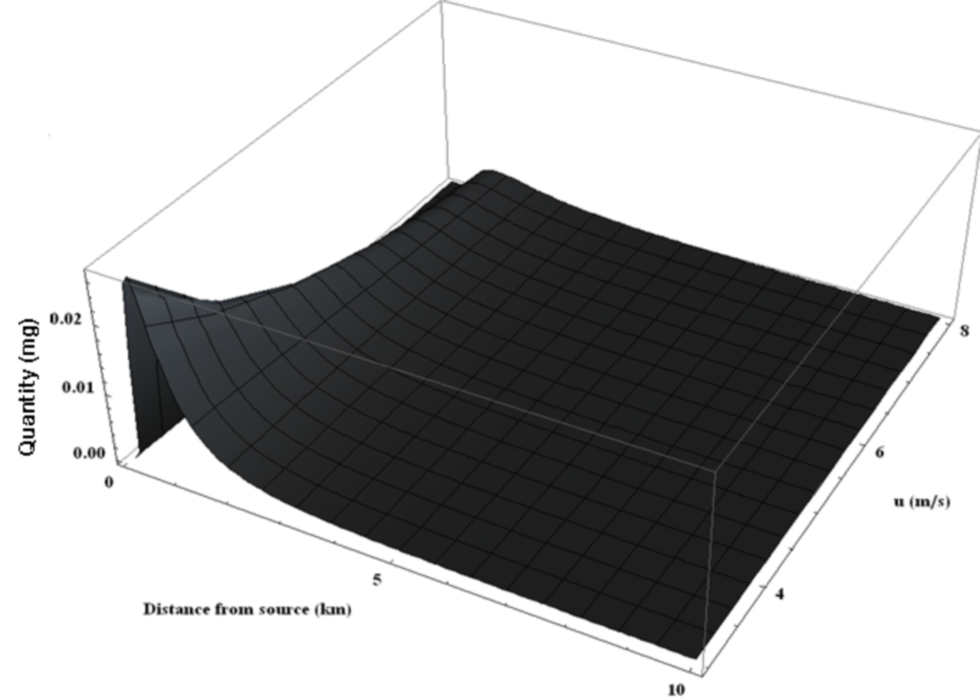

Supplement: Figure S1 — Effect of varying wind speed u on the contaminated dust quantity present on a 4 m square space at various distances from the source at the moment that the deposition arising from a 24 hour-long emission period ends. (TIF) [file pone.0031114.s001.tif]

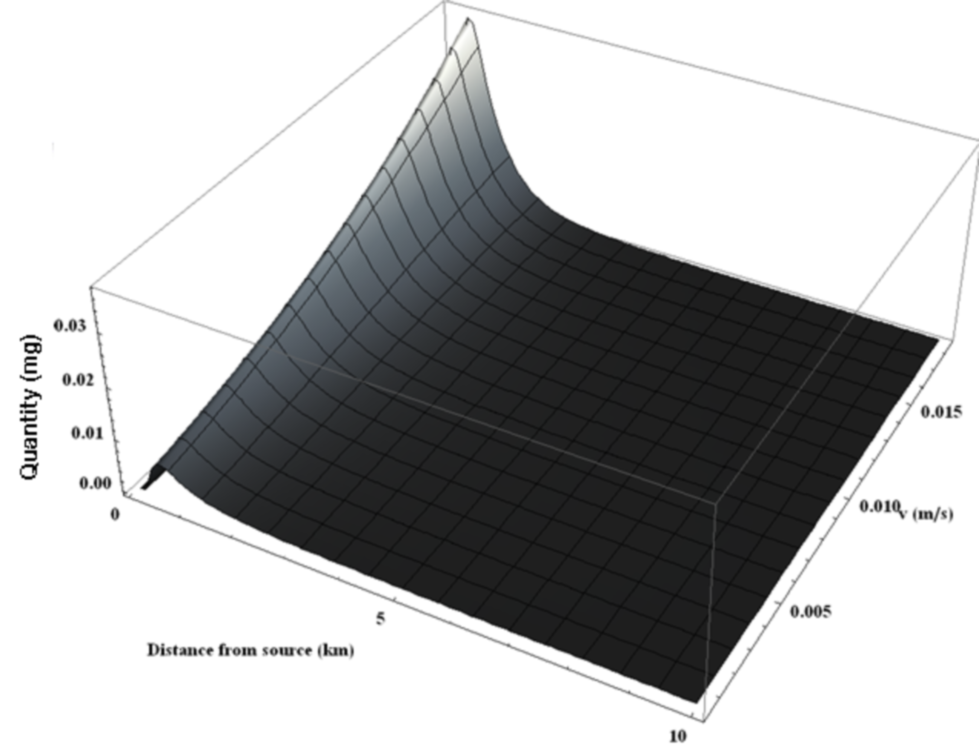

Supplement: Figure S2 — Effect of varying the settling velocity v on the contaminated dust quantity present on a 4 m square space at various distances from the source at the moment that the deposition arising from a 24 hour-long emission period ends. (TIF) [file pone.0031114.s002.tif]

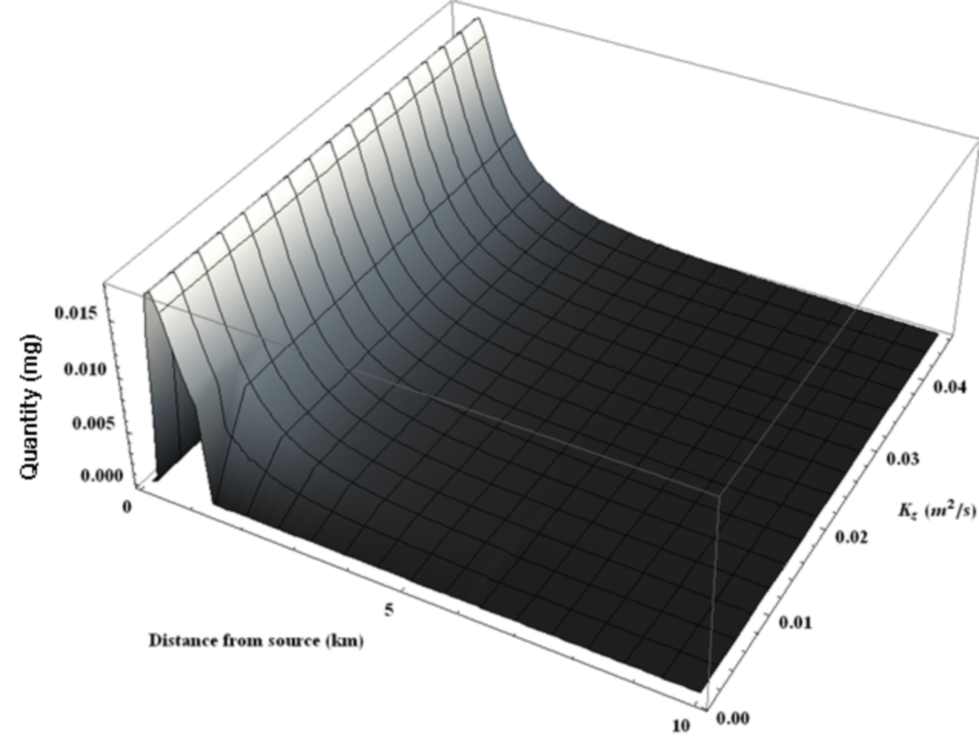

Supplement: Figure S3 — The effect of varying the vertical eddy diffusivity Kz on the contaminated dust quantity present on a 4 m square space at various distances from the source at the moment that the deposition arising from a 24 hour-long emission period ends. (TIF) [file pone.0031114.s003.tif]

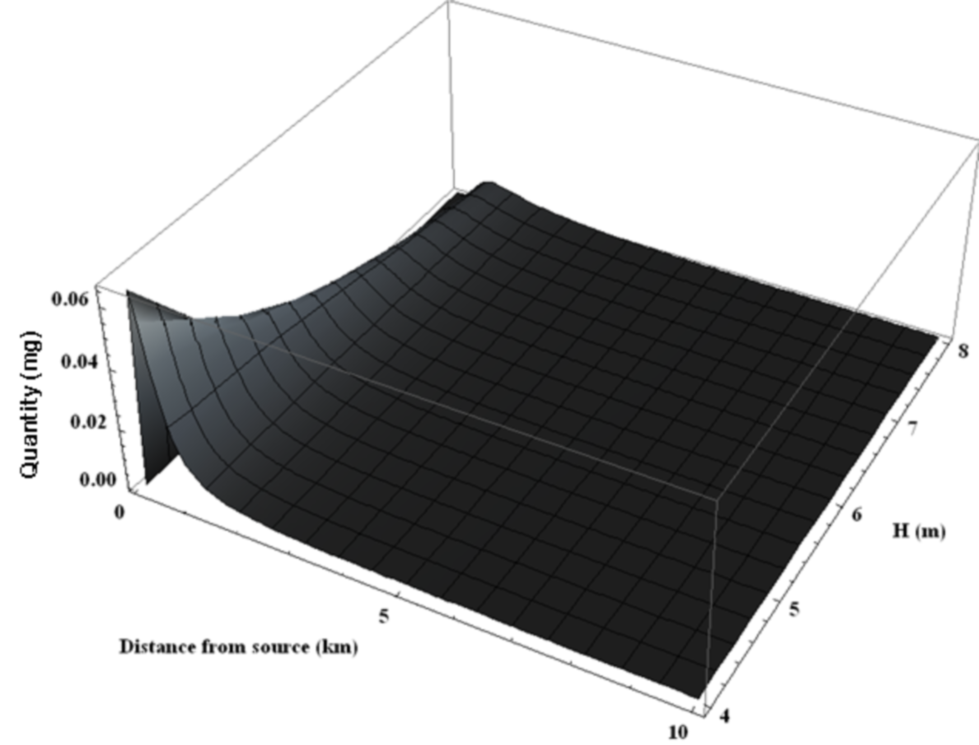

Supplement: Figure S4 — Effect of varying the effective release height H on the contaminated dust quantity present on a 4 m square space at various distances from the source at the moment that the deposition arising from a 24 hour-long emission period ends. (TIF) [file pone.0031114.s004.tif]

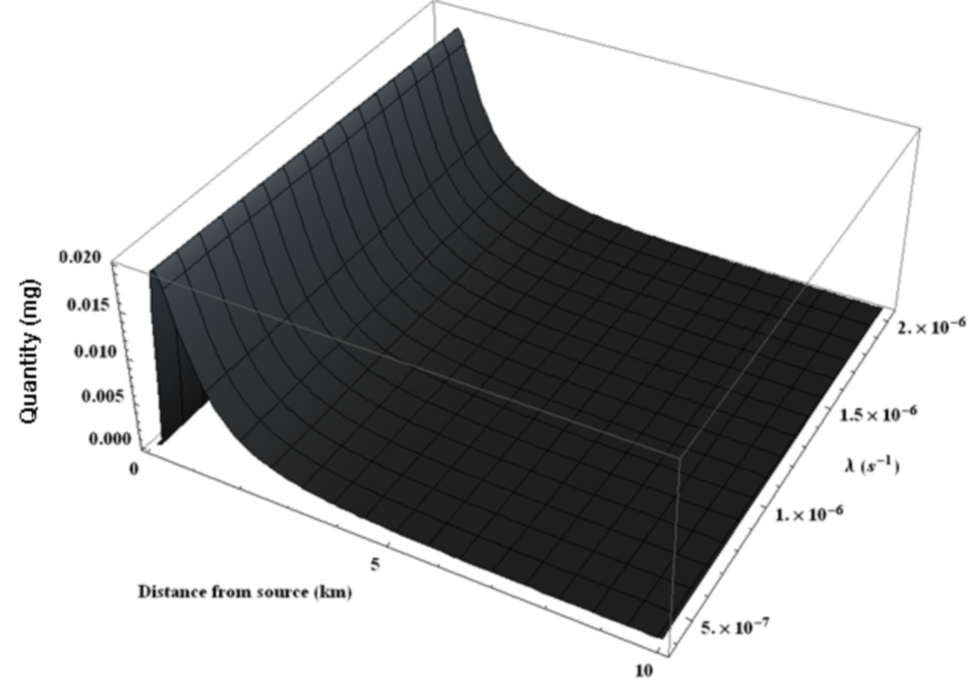

Supplement: Figure S5 — Effect of varying the decay rate on the contaminated dust quantity present on a 4 m square space at various distances from the source at the moment that the deposition arising from a 24 hour-long emission period ends. (TIF) [file pone.0031114.s005.tif]

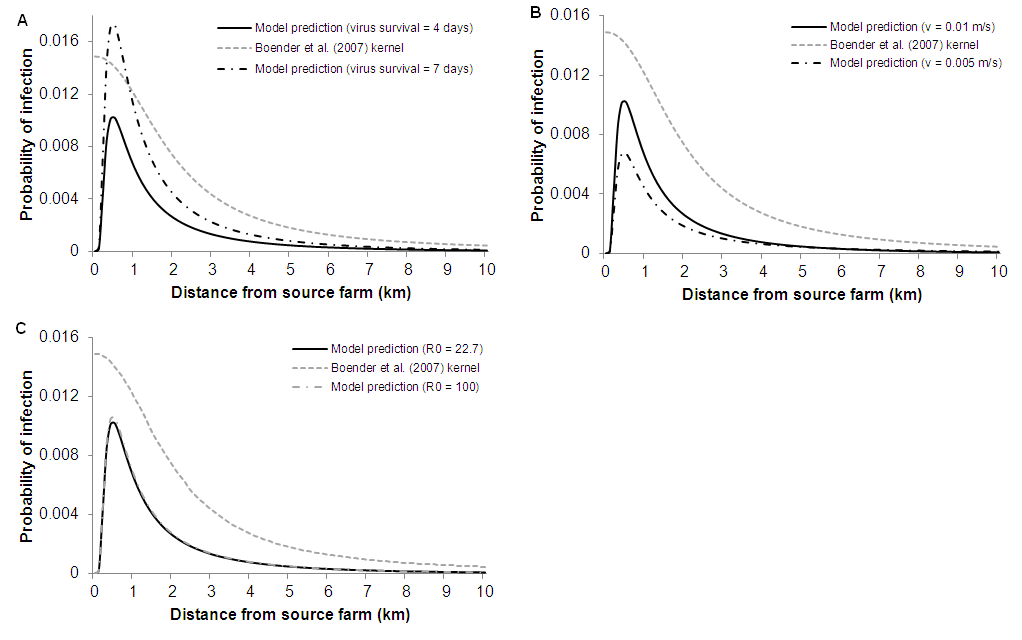

Supplement: Figure S6 — Comparison of the distance-dependent probability of infection as estimated by Boender et al. (2007) from the 2003 epidemic data and our wind-borne spread model prediction with default parameter values and: Panel A. The virus survival was increased from 4 to 7 days); Panel B. The particle settling velocity was reduced from 0.01 m/s to 0.005 m/s); Panel C. The within-flock basic reproduction ratio was increased from 22.7 to 100. (TIF) [file pone.0031114.s006.tif]
